# Marble Waste Dump Yard in Rajasthan, India Revealed as a Potential Asbestos Exposure Hazard

**DOI:** 10.3390/ijerph22020215

**Published:** 2025-02-04

**Authors:** Raja Singh, Sean Fitzgerald, Rima Dada, Arthur L. Frank

**Affiliations:** 1 Department of Environmental and Occupational Health, Dornsife School of Public Health, Drexel University, Philadelphia, PA 19104, USA; alf26@drexel.edu; 2 FACTS, Pllc., Durham, NC 27705, USA; sfitzgerald@fitzacts.com; 3 Laboratory for Molecular Reproduction and Genetics, Department of Anatomy, All India Institute of Medical Sciences, New Delhi 110029, India; rimadadaaiims20@gmail.com

**Keywords:** aravalis, public health, mines safety, asbestos, tremolite, mesothelioma

## Abstract

Asbestos is a fibrous variety of certain minerals, some of which occur naturally as an accessory to a wide variety of mineral resources. Although asbestos itself has been historically mined for various useful properties, the negative health effects of asbestos dust have greatly diminished it as a useful earth material, as many countries have banned the use of these fibrous minerals based on those health concerns. Resulting regulations of asbestos have focused primarily on intentionally mined material used in product manufacturing, such as building materials made with beneficiated asbestos and their derivative exposures, e.g., airborne asbestos in schools with asbestos-containing materials. The hazards of asbestos as unintended byproducts have not been as extensively considered, although this “contamination” has been repeatedly observed in common earth materials including talc, vermiculite, sand, and gravel. This study reveals such contamination of ornamental and dimension stone commonly referred to as “marble”. Asbestos types that can be associated with certain Indian marble reserves include asbestiform tremolite, actinolite, anthophyllite, and chrysotile asbestos. This case reveals such contamination in a marble reserve in Rajsamand, Rajasthan. At this location, marble dust in slurry is disposed at waste collection points, unfortunately including a location now open to the public that has become a tourist destination. Using Transmission Electron Microscopy (TEM) in this study, dust from this location revealed abundant tremolite asbestos fibres in the disaggregated dust. This poses potential health risks to the workers, bystanders, and tourists that may be exposed to this recognized carcinogen, a known cause of mesothelioma, lung cancer, and other asbestos-related diseases.

## 1. Introduction

**Figure 1:**
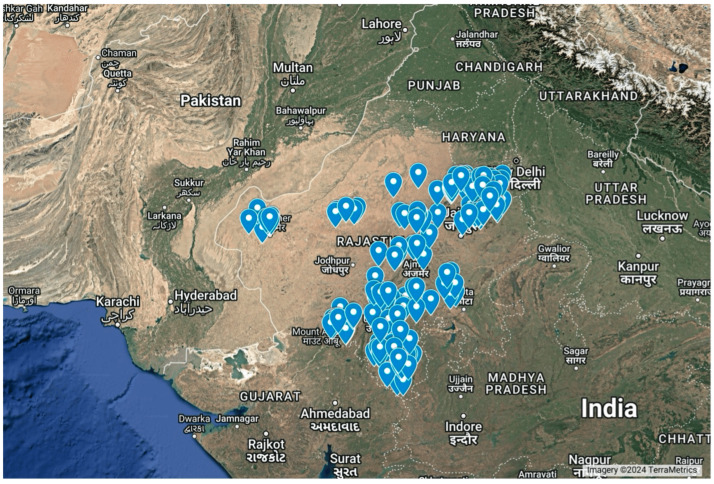
Marble deposits in the state of Rajasthan. Locations published by the Geological Survey of India mapped on Google Earth.

Geologically, marble is defined as carbonate rocks like limestone or dolostone that have been metamorphosed and altered by heat, pressure, and water in the earth, often light-colored with mottling, streaks, and swirls [1]. Slabs and blocks, known in the trades as dimension stone, are frequently used in sculpture and architecture. In India, marble is widely mined, substantially from the state of Rajasthan. A Geological Survey of India report states that the total marble recoverable in India is 1200 million metric tons, of which >90% (~1100 million metric tons) are in Rajasthan [2]. Over 4,270,000 metric tons of marble were produced in Rajasthan in 1999 and 2000 [2]. The location of these marble deposits in Rajasthan from that report is visually represented in Figure 1.

India has a marble standard IS 1130:1969, which classifies marble according to its colour [3]. The plain white marble is sourced from the districts Nagaur, Rajsamand, Sirohi, Jaipur, Alwar, Banswara, and Udaipur. The marble of this region is Precambrian (>540 million years old) and is highly metamorphosed; it is important in that the more extensive the metamorphism from original deposition, the more likely the pressures and temperatures are to produce tremolite as an accessory mineral in the marbles. Further, source limestones and dolomites, when combined with silica and water, have the elemental constituents needed to produce tremolite, such as what we see in the geologic incorporation of silica sands in the metamorphism of these carbonates. In fact, it is well recognized that the metamorphism of siliceous dolomites invariably forms fibrous tremolite [4].

These marble deposits are part of the Bhilwara, Aravali, or Delhi sub-groups. We looked at the Rajsamand District, which has 1637 hectares under marble mining leases, one of the largest in Rajasthan. There are three belts in the Rajasamand District: the Rajnagar-Amet Belt, the Pipali-Kuanria-Dariba Belt, and the Kaliwara-Kumbhalgarh-Charbhuja Belt. The Rajnagar-Amet Belt consists of the Rajnagar-Bhagwandi-Marchanna block [2]. During the extraction and processing of this marble, there is substantial waste marble released as waste marble slurry. It is in the proximity of this block in Rajnagar that there is a marble slurry disposal site, which is described further. As an aside, marble waste slurry disposal has been reported to be harmful to the environment by another study conducted by the Geological Survey of India in Makrana (Nagaur) in Rajasthan [5]. That report shows rills and gulleys developed over dumped marble wastes. These surface runoff washouts are likely sources for groundwater and surface soil contamination observed in the areas surrounding the waste dumps. Calcium, SO_4_, NO_3_, and Magnesium were found in soil samples. There were high Total Dissolved Salts (TDS) and SiO_2_ in the water samples, which were derived from the mine water pit and the water which was laden with the marble slurry [5]. Although the general assessment of the effects of marble mining in the report shows a positive social economic improvement, the overall environmental and health cost is potentially substantial, especially in the long term. Regarding the environmental damage, in a report on Makrana marble by the Geological Survey of India, severe geoenvironmental degradation was reported in the area. Due to seepage, the ground water was contaminated due to the release of wastewater in open spaces, and the conditions in one study showed that the water table dropped below the normal depth and has reached 40–50 metres below ground level due to mining. Water in the area also shows high pH, TDS, Ca, Mg, SO_4_, NO_3_, Na, K, Cl, HCO_3_, and SiO_2_. The total impact score was recorded at “−3875”, which is an impact level regarded as

‘Major injurious impact on the environment. Major environmental control measures to be taken and/or site selection for the proposed project to be reconsidered within the buffer zone [5]’.

In 2023, there was a case before the National Green Tribunal regarding the marble slurry being disposed of out of the designated areas in Rajsamand, Rajasthan [6]. Upon the complaint and on the intervention by the local authorities, the slurry was removed, though the method of removal, remediation, and dust control monitoring was not clear. With heavy rainfall, some slurry went outside of designated areas. Also, slurry has been given to local residents for use as construction material, often used by local masons and craftsmen, which may usually be without protective equipment or awareness training.

Guidelines for the management of marble waste slurry have been developed by the CPCB or the Central Pollution Control Board of the Government of India, which have focussed on the disposal of marble slurry waste in designated disposal spots. The use of marble slurry as a construction material, in cement manufacturing, in synthetic gypsum production, in road construction, and in tile manufacturing has also been promoted by the state pollution board of Rajasthan and by the CPCB [7,8]. Many studies have been conducted of these not fully considered applications [9,10,11,12,13,14]. Regardless, the current solution is the disposal in designated waste collection spots, which often translates to very large collections in some places. Although these open sites may be against environmental and health norms, there may be little to no enforcement of hygiene or standardized procedures for the handling of these materials.

**Figure 2:**
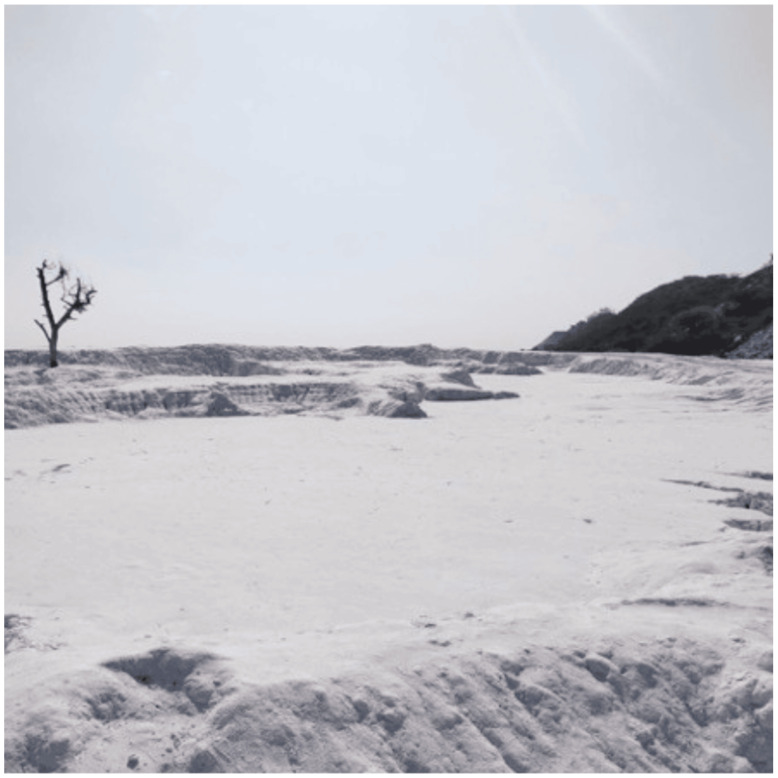
Photograph of the Marble Slurry Waste collection spot near Mokhampura, Kelwa in District Rajsamand, Rajasthan. (Source: Praveen K, Authors.)

One such site in Rajasthan, i.e., the marble slurry waste deposit spot located in Kelwa in the Mokhampura area or the Rajsamand District, is not only a marble slurry waste deposit site but has also become a tourist spot in Rajasthan (see Figure 2). In fact, Rajasthan Tourism promotes this location as a tourist spot with a description: *“Discover the captivating allure of Rajsamand Dumping Yard! With its vast expanse of white marble slurry, this hidden gem provides a breathtaking backdrop for memorable photoshoots.”* [15]. It has also been reported that photographers bring in wedding couples for photoshoots at this location in Rajasthan [16]. The site even sells entry tickets and facilities that are being provided by the marble manufacturers association. This is regardless and inconsiderate of the potential negative human health impact, as marble slurry is an environmental waste and hazard that contains constituents likely to affect the people who may be exposed to the dust, who are being encouraged rather than discouraged to disturb this potentially hazardous dust and debris, also carrying it out through car and person movement, which dries and becomes airborne. This should stop being promoted as a tourist and photographic venue. Because of asbestos contamination, interaction with such dust should be kept to a necessary minimum, require training and awareness and involve the use of personal protective equipment under strict regulations [17].

In the dry season, the sludge dries up, mixes with wind, and deposits on the vegetative landscape. The main issue is the impact of marble slurry not only on the environment, which has been demonstrated in studies, but the potential impact on human health [5]. The slurry, when dried through wind or movement in rains to other areas and subsequently drying, and when used by local residents as construction material, can cause health effects from dust made airborne. Further, it had been reported by the Geological Survey of India in the past that contractors dispose of slurry in nearby open lands during the night. During rains, this slurry spreads; then when dry, it pollutes the air and travels far beyond the confines of the disposal fields. There is also high air and noise pollution (due to the use of machinery) and the dust deposits on flora and fauna [5].

These health effects can be substantial when the marble has natural contamination with asbestos, which can include chrysotile, tremolite, or actinolite, based on the local geology. In the Rajnagar marble area of Rajsamand, where the marble slurry waste disposal in this current study is concerned, the petrographic analysis by the Geological Survey of India states that the marble contains high silica, actinolite, tremolite, and diopside [2]. Actinolite and tremolite are two identified forms of asbestos when fibrous, which are two of the six types of asbestos that are regulated as a known health hazard. Asbestos is the collective term used for six types of minerals that have been classified into two categories, amphiboles and serpentine. Chrysotile is serpentine asbestos, and the amphiboles regulated when fibrous are actinolite, tremolite, crocidolite, amosite, and anthophyllite. All varieties of asbestos are carcinogenic, and their inhalation can cause asbestosis and malignancies like mesothelioma or lung cancer [18]. Asbestos silicate fibres in lung tissue also leads to epigenetic changes by inducing oxidoreductive reactions on the surface of these fibres which not only induce DNA damage, but also induce de novo mutations and epigenetic alterations. Previous studies have documented that oxidative stress induces global hypomethylation and especially the loci-specific hypermethylation of tumour suppressor genes [19]. This is the underlying aetiology and main driver for cancers. This will also aid in directing more effective therapy against these epigenetic alterations. Chrysotile and crocidolite types of asbestos induce inflammation and oxidative stress, and there is an extensive alteration in the expression of the genes. These are genes involved in cell cycle regulations, DNA repair, and integrin mediated signalling [20].

It may be noted that petrographic analysis may not provide an indication of a direct health hazard, as the ingrained asbestos fibre may not have been released. Hence, it is important to understand whether there is a release of asbestos fibres from the marble when it is extracted, cut, finished, or transported. In this study, the dust from the marble slurry located near Mokhampur, in Kelwa, District Rajsamand, Rajasthan, has been analysed to check for the presence of asbestos.

The image of the marble slurry waste deposit spot located in Kelwa in the Mokhampura area or Rajsamand District, which is not only a simple waste deposit site but has become a tourist spot in Rajasthan, is shown in Figure 2 below.

Aim: The study aims to check the presence of asbestos in the marble slurry dust that finds its way into the open environment and to develop appropriate testing protocols and suggest awareness training programs when asbestos from these potential sources is found.

A sample of slurry waste dust sampled from the open ground of the designated waste collection point was collected in December 2023 and analyzed in January of 2024. The slurry sample was analyzed for constituent characterization, identification of minerals, and to determine the presence of asbestos detectable by Polarized Light Microscopy (PLM) and Transmission Electron Microscopy (TEM), as prescribed in the Test Method EPA/600/R-93/116: Method for the Determination of Asbestos in Bulk Building Materials published by the Environmental Protection Agency of the United States [21].

Further, as the material as a disaggregated dust is likely to make respirable-sized particles airborne, AHERA (The Asbestos Hazard Emergency Response Act, 1986 of the United States of America) counting rules were all used in the TEM analysis to further analyze the material to see if asbestos structures countable by that method were present, and if such were found, they were quantified on a structures-per-gram weight basis (asbestos str/g of slurry soil dust) [22].

Polarized Light Microscopy (PLM) analysis of the material was first conducted using a Leica DM750P petrographic microscope with wave retardation, cross-polarized filters, and dispersion staining techniques at magnifications up to 400X. Transmission Electron Microscopy analyses were conducted on a JEOL 2000FX (up to 50,000x, 120KeV) equipped with Energy-Dispersive X-ray Spectroscopy (EDS) for chemistry and structure by Selected Area Electron Diffraction (SAED).

The preparation for electron microscopy analysis was carried out by weighing and suspending a portion of the sample in a water and alcohol mix. The suspension was prepared in a manner consistent with ASTM D6480 Standard Test Method for Wipe Sampling of Surfaces, Indirect Preparation, and Analysis for Asbestos Structure Number Surface Loading by Transmission Electron Microscopy, i.e., aliquots of the measured volume of the sample suspension followed by filtration through a 0.2 µm mixed cellulose ester (MCE) filter paper [23]. The final MCE filter was dried, acetone-collapsed, and coated with carbon in a vacuum evaporator. The solids and fibres which were collected on the carbon-coated filter replicate were transferred onto copper grids for TEM analysis.

## 2. Results

**Figure 3:**
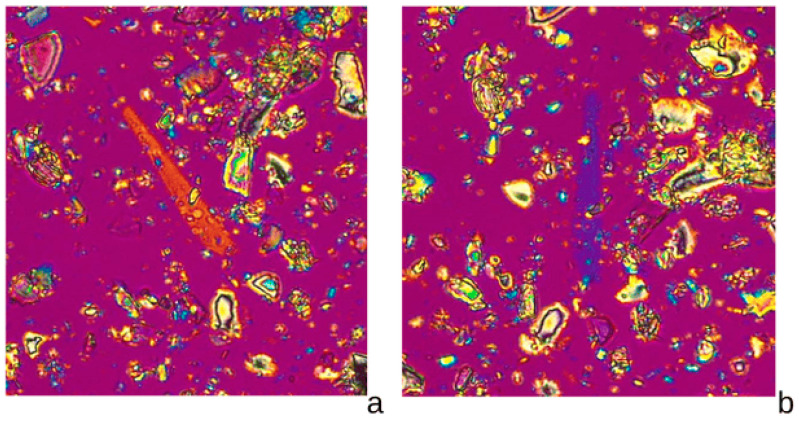
PLM Identification of elongate to fibrous tremolite constituents of the Kelwa dust sample. The fibre-bundle rotated (**a**,**b**) to show signs of elongation and oblique extinction characteristics consistent with tremolite, with 200× magnification, cross-polarized light, and a 430 nm wave plate compensator. Particles in the Kelwa dust sample were also found to be consistent with other diagnostic optical characteristics for tremolite, including birefringence and refractive indices, measured in 1.605 HD RI oil particle mounts. HD: High Dispersion; RI: Refractive Index; Source: Authors.

The analysis of the Kelwa marble dust for mineral content and possible asbestos by only PLM did not conclusively detect fibres consistent with asbestos at the light microscope level of detectability. However optical characteristics consistent with the mineral tremolite were observed (Figure 3).

**Figure 4:**
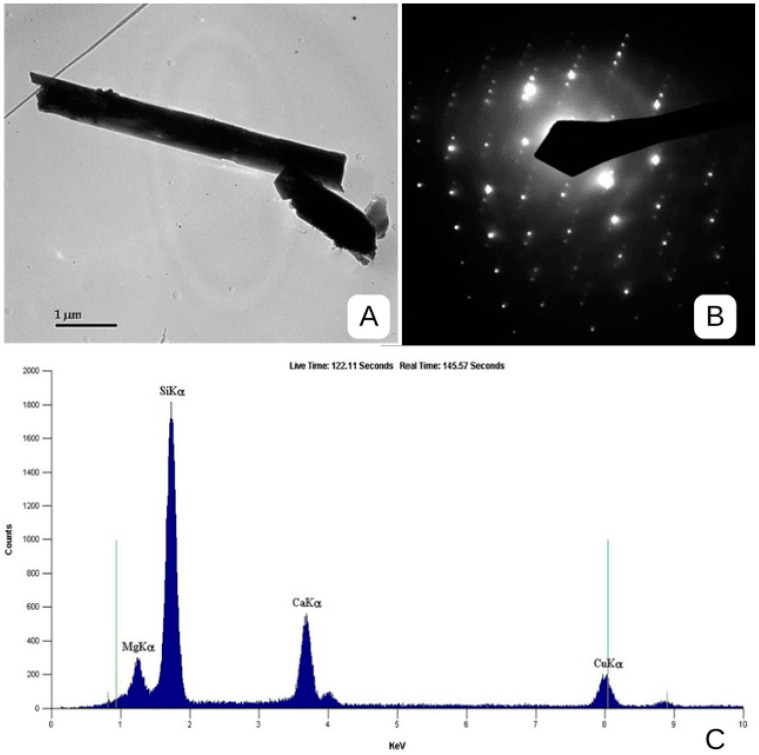
Photomicrograph (**A**), diffraction pattern or SAED (**B**), and chemistry or EDS of the tremolite structure (**C**), 5.4 µm long containing fibres 0.16 µm wide, bundle width of 0.53 µm wide, identified in this testing (Marble Dust from Kelwa, Rajasthan, India). SAED: Selected Area Electron Diffraction; EDS: Energy Dispersive X-ray Spectroscopy; Source: Authors.

**Figure 5:**
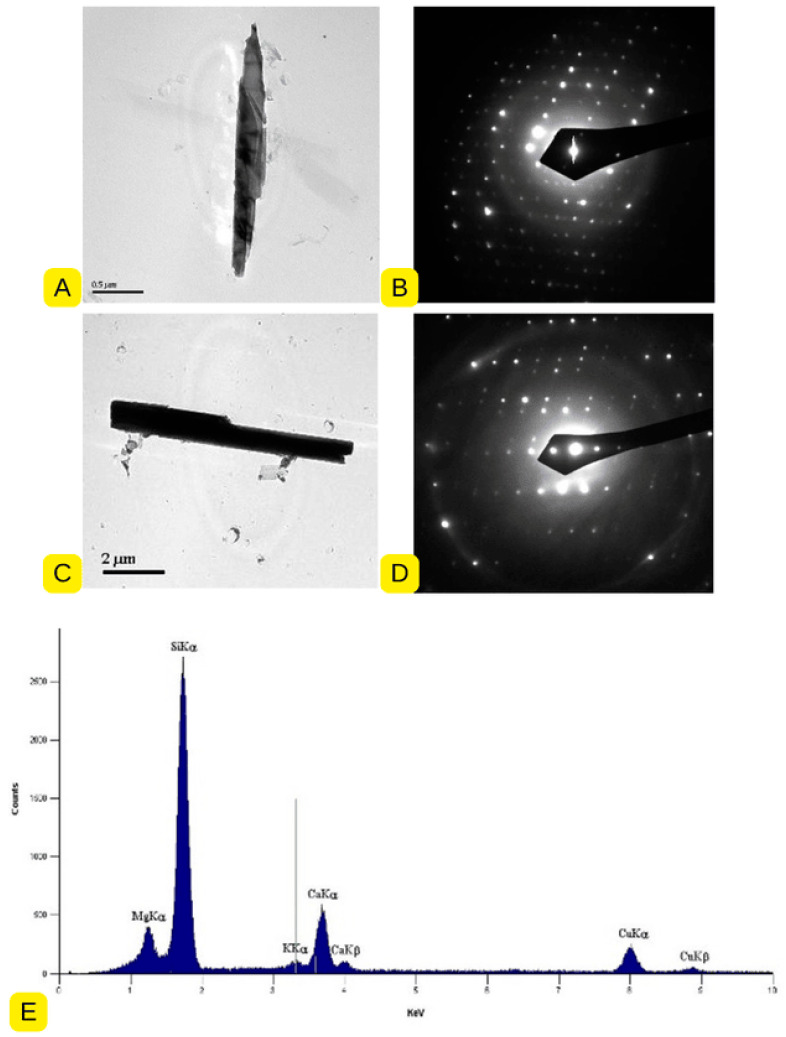
Photomicrograph (**A**,**C**), diffraction pattern or SAED (**B**,**D**), and chemistry or EDS (**E**) of some more countable tremolite structures identified in this testing (Marble Dust from Kelwa, Rajasthan, India).

Subsequent analysis by TEM confirmed tremolite mineral chemistry by Energy dispersive X-ray spectroscopy or EDS, and the structure by Selected Area Electron Diffraction or SAED. Tremolite particles were found abundantly dispersed throughout the material, in morphologies ranging from blocky, bladed and euhedral, with most tremolite particles less than 10 microns, preferentially oriented to elongate, with a substantial constituent identifiable as fibres/fibrous structures, which can be counted as asbestos structures (See Figure 4 and Figure 5). According to the AHERA protocol, these would be considered countable and, by size, inhalable.

The tremolite asbestos concentration measured in fiber structures per gram (str/g) of the dust was calculated at 11,800,000 str/g for the size fraction > 5.0 µm long and 37,100,000 str/g in consideration of all AHERA-countable asbestos fibre-structures. Adding in all tremolite particle sizes and shapes bumps the tremolite structure count to 103,000,000 str/g.

**Figure 6:**
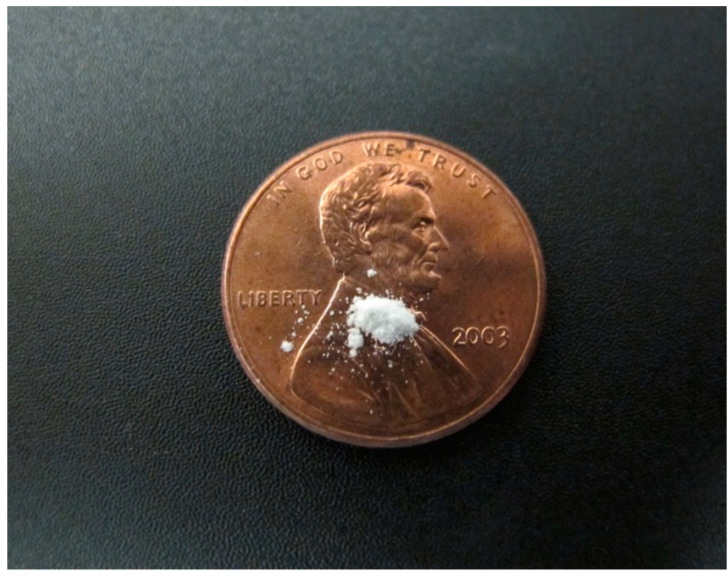
Ten milligrams of talc for scale on a U.S. penny [24]. An equivalent amount of the Kelwa Marble Dust, as tested, would contain approximately 37,000 countable fibrous structures of the regulated asbestos mineral tremolite. Source: Authors [24]. U.S.: United States.

Ten milligrams of Kelwa Marble Dust, as tested, would contain approximately 37,000 countable fibrous structures of the regulated asbestos mineral tremolite (See Figure 6).

## 3. Discussion

The initial impetus for this study was a study reported from Udaipur in Rajasthan which documented 76 cases of mesothelioma in 4 years from one hospital [25]. Surprisingly, all of the subjects denied any past exposure to asbestos. However, 69 out of the 76, or 91%, reported having history of direct or indirect exposure to the mining and quarrying industries of marble and granite. That study did not investigate asbestos’ presence in marble mines but noted that *‘asbestos is present in close association with marble in rocks and quarrying might lead to airborne asbestos fibres which may be implicated as a causative factor in this region’* [25]. All of the Udaipur subjects were from a marble mining area of Rajasthan. The current study bridges this information gap by actually testing the marble dust and confirming the presence of tremolite, a type of asbestos which is an etiological factor for mesothelioma. Further, other studies report that protective equipment and precautions are seldom used in mining in Rajasthan, with little or no health record-keeping with workers who are generally in the unorganized sector [26,27].

Studies from other parts of the world were also reviewed. In Namibia, the Kribib area has commercial marble called Rhino White Marble. Amphibole asbestos, e.g., tremolite, has been observed in these metamorphic rocks. During the extraction or quarrying of the marble, tremolite can get released into the air [28]. Importantly, the United Kingdom’s Health and Safety Executive studied marble samples and reported the presence of tremolite fibres in the majority of the samples that were studied; however, their advisory documents stated that the tremolite may be in a low quantity of the total volume of the marble and that only during cutting or grinding may the asbestos be released [29].

In a study from the U.S., which called for a policy on naturally occurring asbestos, clusters of mesotheliomas were analyzed through a literature review in places where direct daily exposure to naturally occurring asbestos was common [30]. This included agricultural villages of New Caledonia, Greece, Turkey, Cyprus, and Corsica, where the white soil containing tremolite asbestos is commonly used in white stucco for whitewashing walls or to plaster them [31].

There have been other studies regarding dimension stone and possible asbestos exposure from Italy; however, the Italian studies dealt with the presence of chrysotile in Valmalenco serpentinites, but the maximum exposure of asbestos released into the air was near the block cutting, which seems to have used multiple blades. The second was at the drilling sites in the quarries [32]. In another study on green “marble” or serpentinite, called Malenco Serpentinite, it was reported that small amounts below 400 ppm were detected in soils and stream sediments along with traces of asbestiform tremolite. Due to slip-fiber mineralization, a chrysotile layer was expected, but the tremolite was attributed most likely as originated from the talc veins and/or the ophicarbonates (or serpentinites containing carbonate minerals in appreciable amounts) that occurred both in serpentinites as well as dolomitic marbles [33].

As to the releasability of asbestos, it may be stated that even low asbestos concentrations may mean a potential human health risk [34]. The U.S. Environmental Protection Agency (EPA) and the Agency for Toxic Substances and Disease Registry (ATSDR) have studied several sites where low asbestos contents produced airborne concentrations of significant concentrations, including sites where asbestos was naturally present [35,36]. Therefore, even fractions of a percent of asbestos content by weight leads to proven substantial airborne concentrations, which is also the case for the marble “dust” or soil tested here. There has been a debate on this due to the issue of a recent creation of a release index developed in Italy, which creates a process where a standard quantity is ground to check the actual releasability, but that particular study used the serpentinite, as opposed to the current study, which uses the actual dust on the ground after release, which shows a positive asbestos content [37]. Also, mesothelioma was reported from clusters in excess where natural asbestos fibres were found [38].

In Korea, there has been some debate and discussion on the regulation of construction stones which may contain naturally occurring asbestos. Dolomite from a mine in Korea contained asbestos, as well as the talcum powder for babies available in the Korean market. The serpentinite from Korea also contains chrysotile. Korea has a ban on asbestos and there is an Asbestos Safety Management Act passed in 2012. But, due to the commercial importance of construction stones, the Korean Ministry of Labour and Employment changed the standard for the content of asbestos in materials from 0.1% to 1% to create harmony with the occupational safety law. But it is generally important to consider the naturally occurring asbestos in construction material (apart from materials that have intentionally added asbestos/industrial products) when the regulation of asbestos is concerned, as occupational exposure from naturally occurring asbestos is possible, especially considering cases of occupational asbestos exposure in Korea increased each year [39].

The lack of regulation, apart from the effect on health and the environment, can also mean economic loss to marble miners. Due to regulations in Australia, the import of asbestos containing stone ‘knowingly’, even with trace levels, is considered a serious offence under the Customs and Workplace Health and Safety (WHS) Laws. It is also reported that Indian Bidasar varieties contain asbestos, which may have been imported in Australia from India [40,41]. This means that having proper testing and monitoring of Indian stone is not only important for the sake of occupational health and safety but also to prevent trade prohibitions for Indian materials.

The under-reporting of exposure is a big concern, as there can be brief unknown and para-occupational exposures. A short period of indirect exposure during childhood is often ignored or unknown, leading to an under-reporting of the cases or understanding of the dose-response or aetiology (cause of disease) [42]. This may particularly be aggravated when there is a lack of awareness about the presence of asbestos in a material or where such information may not be easily available or circulated. This is more likely than not the case with asbestos fibres in marble, which is mined in Rajasthan and elsewhere in India.

Asbestos in India is widely used in the form of building products, where chrysotile fiber is imported and processed in India [43]. There are also major concerns with the non-occupational use of asbestos cement sheets, where the end users may be exposed to asbestos fibres due to the weathering, demolition, renovation, and disposal of asbestos cement roofing sheets [44]. Asbestos has also been reported in commercial talcum powder in India, which means that millions of people may be exposed to asbestos, and this aetiological factor for asbestos-related diseases may never occur to physicians [24,45,46]. There are also ship breaking and recycling industrial operatives in India with large numbers of mesothelioma cases predicted [47]. Apart from the study from Udaipur, where 76 cases were reported and possibly linked to asbestos-contaminated mining, there have been large studies on mesothelioma in a single hospital in Gujarat [25,48]. This hospital in Gujarat receives several patients from Rajasthan [48]. Generally, with only 16% of the Indian population under the National Cancer Registry Program, many hospitals are not recording cases in a centralized registry, likely responsible for under-reporting of mesothelioma cases [49]. This is of much concern, as it is predicted that there will be at least 12.5 million patients with asbestos-related disease in the near future. Further, it is predicted that there will be 1.25 million patients with asbestos-related cancers. In this worldwide cancer figure, half may be from India itself [50]. Under these circumstances, looking at the incidental exposure in mines with asbestos contamination needs urgent review to prevent asbestos-related diseases in India.

## 4. Conclusions

Asbestos-related diseases are significant in India and have been seen to be on a rise across the world. This study aimed to determine whether the waste slurry from a marble waste slurry dump contains detectable asbestos fibers. It has been reported in this study that the dust from a marble slurry dumping yard open to the public as a tourist location has been found to contain tremolite asbestos. This plausibly demonstrates that the marble industry poses an occupational and environmental hazard to the workers, local inhabitants around the mine, and the general public at a distance by exposing them to asbestos dust. It is well recognized in the scientific and medical community that such exposure may lead to asbestos-related diseases, which can include lung cancer or mesothelioma of the pleura or the peritoneum. This informs us that during the extraction process, or during transportation, refining, or any other process in and around these marble mines, the tremolite can be released into the environment, into the breathing zone of not only the workers but the bystanders or even tourists. This is crucial, as there may not be general awareness about the presence of asbestos contamination in marble widely extracted in Rajasthan, and physicians, workers, or mine owners may never be aware of asbestos exposure from “marble” mines or milling units.

Recommendations include the following:

There should be wide-scale monitoring and testing of the inhalable dust that is released in marble and dimension stone mining in Rajasthan and other areas so that particular lots of the stone can be marked and extra precautions can be taken.

Additionally, there should be considerations for general dust precautions; worker protective equipment should be used, but now, the personal protective equipment and other dust control methods in mines should be made compulsory and such actions should be monitored and regulated by government agencies [51]. This is especially needed for marble mines, marble processing areas, marble installation sites, and construction sites, all of which should fall under studies designed to determine background and episodic airborne values based on task.

Cancer should be made notifiable throughout the country in India, as migrant labor may come from states where cancer may not be notified. In particular, mesothelioma, a malignancy almost always caused by asbestos, should be more thoroughly determined, reported, and tracked.

This study should further encourage the consideration of a policy in India for naturally occurring asbestos to be identified in all types of stone where asbestos may be present so that the miners, workers, and inhabitants in such areas can gain access to awareness and protective measures to reduce exposure. Occupational physicians, pulmonologists, and oncologists could then also include this geographical data in history-taking and may be aware of the possibility of asbestos-related disease when a worker is diagnosed without possible exposure, e.g., from previously thought non-asbestos mining activity.

As per the Indian legislation called the Mines Act, 1952, rules called the Mines Rules, 1955 have been framed. In Section 29B, these specify initial and periodical medical examinations for persons employed in a mine. For general mines, the periodicity of testing is once in five years, but for persons ‘engaged in the process of mining or milling of asbestos ore’, the periodicity is once per twelve months, with the X ray conducted at more frequent intervals in case dust-related disease is needed to be confirmed (compared to the X ray being conducted every three years normally in these cases) [52,53].

The marble slurry designated waste deposit location should stop being promoted as a tourist and photographic venue. Interaction with such dust should be kept to a necessary minimum and require training, awareness, and personal protective equipment.

When marble is used in homes and buildings, the workers and residents may be exposed during cutting, polishing, and grinding. Awareness is needed and proper guidelines are to be framed for precautions for workers and residents during marble installation. Since marble dust is used for tile manufacture, the same precautions during tile installation may need to be in place. This may include the use of personal protective equipment and the prevention of the spread of dust to other areas in the proximity of the construction site. If airborne asbestos fibers are plausible in the work, there should also be prescription to change work clothes before reaching home for workers to prevent para-occupational exposure for the partners and family of the workers.

Compensation and social security for miners in mines containing naturally occurring asbestos must be treated at par with asbestos industry workers (because the mining of asbestos is banned in India and the industry regulations may be relevant,) and the directions given by India’s apex judicial body, or the Supreme Court of India, must be followed, as in the Consumer Education and Research Centre judgment of 1995, by adapting the same for miners [54,55]. The adapted recommendations for mines having asbestos as a contaminant and the facilities where the milling of such minerals happens, in line with the judgement for asbestos industries, may be as stated further.

The health records of the workers in the mines for a minimum period of forty years should be maintained. Or, the health records fifteen years after retirement or the end of employment should be maintained, whichever may be later.

Mines with naturally occurring asbestos may undertake membrane filter tests, or its equivalent, to detect fibres of asbestos. Even though these rules already may exist for asbestos mines partially or in full, these must be extended for mines which are of minerals that have asbestos as a contaminant, like marble in some areas, and must be implemented on the ground with thorough monitoring. Further, the permissible limit of asbestos fibres for mines having asbestos as a contaminant (asbestos fibres limit of 1 fibre per ml) should be made a more stringent limit, as in factories, where it is is 0.1 fibre/cc [56,57]. However, no amount may be considered safe, and the International Labour Organization states that there is no safe way to handle asbestos [58,59,60].

All mine workers should be covered under the Employee State Insurance Act or its equivalent, if not already covered.

The standards for the permissible exposure limit to asbestos must be reviewed as per the International Labour Organization (ILO) standards as and when they are released. States should only approve mine leases if, as a pre-approval step, the mines compulsorily notify, under the Mines Act, 1952, to the Directorate General of Mine Safety, which is run by the Central Government. This calls for enhanced coordination between the Centre and the states in India, with the Centre, by the Constitution of India, being given the responsibility of mine safety and the state being given the mine lease revenue collection power [61].

## Data Availability

Data is contained within the article. Further inquiries can be directed to authors. The work has been posted as a preprint on medRxiv in October 2024 [62].
